# Dihydromyricetin improves growth performance, immunity, and intestinal functions in weaned pigs challenged by enterotoxigenic *Escherichia coli*

**DOI:** 10.3389/fvets.2024.1421871

**Published:** 2024-07-26

**Authors:** Kunhong Xie, Jiawen Qi, Lili Deng, Bing Yu, Yuheng Luo, Zhiqing Huang, Xiangbing Mao, Jie Yu, Ping Zheng, Hui Yan, Yan Li, Hua Li, Jun He

**Affiliations:** ^1^Institute of Animal Nutrition, Sichuan Agricultural University, Chengdu, China; ^2^Key Laboratory for Animal Disease-Resistance Nutrition of China Ministry of Education, Chengdu, China; ^3^College of Veterinary Medicine, Sichuan Agricultural University, Chengdu, China

**Keywords:** *Escherichia coli*, immunity, DMY, intestinal epithelium, microbiota, weaned pigs

## Abstract

Enteric infection is a major cause of enteric disorder in neonatal pigs during the weaning transition. Dihydromyricetin (DMY) is a natural flavanonol compound extracted from *Ampelopsis grossedentata* with numerous biological activities such as antioxidative and immunomodulatory functions. The objective of this study was to investigate the effects of dietary dihydromyricetin supplementation on growth performance, immunity, and intestinal functions in weaned pigs challenged by enterotoxigenic *Escherichia coli* (ETEC). In total, 24 weaned DLY (Duroc × Landrace × Yorkshire) pigs were allotted to 3 treatments. Pigs fed with basal diet or basal diet containing 300 mg/kg DMY were orally infused with sterilized culture or ETEC (2.5 × 10^11^ colony-forming units). Dietary DMY supplementation significantly elevated the final weight and average daily gain (ADG) but reduced diarrhea incidence in the weaned pigs of the EDMY group compared to the pigs of the ECON group (*p* < 0.05). Compared to the ECON group, DMY also improved the digestibility of dry matter (DM), ether extract (EE), gross energy (GE), and ash of the EDMY group (*p* < 0.05). Moreover, DMY not only significantly decreased the ratio of albumin/globulin but also elevated serum concentrations of immunoglobulins (e.g., IgA and IgG) in the weaned pigs of the EDMY group compared to the pigs of the ECON group (*p* < 0.05). Interestingly, the villus height, the ratio of villus height to crypt depth (V:C), and the activities of mucosal alkaline phosphatase, sucrase, and maltase in the duodenum and jejunum of the EDMY group were higher than those in the ECON group (*p* < 0.05). Importantly, DMY significantly elevated the expression levels of jejunal zonula occludens-1 (ZO-1), claudin-1, cationic amino acid transporter-1 (CAT-1), and fatty acid transport protein-1 (FATP-1) in the weaned pigs of the EDMY group compared to the pigs of the ECON group (*p* < 0.05). Additionally, compared to the ECON group, DMY increased the concentrations of microbial SCFA metabolites (e.g., acetic acid and propanoic acid), but reduced the abundance of *Escherichia coli* in the cecum of the EDMY group (*p* < 0.05). Dietary DMY supplementation can attenuate the ETEC–induced growth retardation and intestinal injury, which was attributed to the amelioration of intestinal nutrient digestion and transport functions as well as the improved microbiota.

## 1 Introduction

The weaned pigs lack effective resistance to diseases and stress due to the deprivation of maternal passive immunity and incomplete gastrointestinal (GI) tract development (1). Previous studies indicated that pathogenic bacteria can induce diarrhea, growth retardation, and even considerable mortality in piglets, especially in the first week after weaning, which causes huge economic losses in pig farming (2, 3). Enterotoxigenic *Escherichia coli* (ETEC), as the most common and typical infectious pathogen of postweaning diarrhea (PWD) for piglets, is characterized by various adhesions [e.g., F4 (K88^+^), F5 (K99^+^), F18, and F41 fimbriae], which could promote malignant colonization on the intestinal mucosa via specific fimbriae receptors (4). During ETEC-induced diarrhea, the intestine is subjected to enormous stress, accompanied by remarkable changes in microbiota homeostasis, epithelial brush border enzymatic activities, intestinal permeability, and intestinal morphology, such as villous atrophy and crypt hyperplasia (5, 6). In past decades, antibiotics have been widely used as therapeutic drugs for ETEC infection to prevent diarrhea and intestinal injury in pig production. Nevertheless, the abuse of antibiotics can lead to bacterial resistance and drug residues in animal products, eventually threatening human safety (7). Therefore, alternatives to antibiotics that are safe for humans, animals, and the environment are urgently needed. Currently, some plant-derived compounds are reported to improve intestinal function, barrier, and microbiota dysbiosis, deeming they are a good choice for antibiotic substitutes (8–10).

Dihydromyricetin (DMY, 2R,3R-3,5,7,3′,4′,5′-hexahydroxy-2,3-dihydroflavonol), as a natural flavonoid compound mainly extracted from *Ampelopsis grossedentata* (*A. grossedentata*), has received considerable attention for its several biological properties such as anti-inflammatory (11), immune-enhancing (12), and intestinal microbiota-regulating biological activities (13). Recently, DMY has also been found to inhibit the replication of some viruses such as the African swine fever virus *in vitro* (14). These advantages suggest a promising protective role of DMY in preventing intestinal injury in animal production. For growing-finishing pigs, dietary supplementation of DMY can effectively ameliorate the intestinal barrier by increasing the immune compounds such as immunoglobulin M (IgM) and IgA contents (12). In addition to this, a previous study has shown that DMY significantly depressed the inflammatory response and restored the intestinal redox status, thus attenuating ileum injury in chickens that suffer *E. coli* lipopolysaccharide infection (15). Intriguingly, it has been reported that the water extract of *A. grossedentata* can regulate gut microbiota, promoting the growth of beneficial bacteria (e.g., *Lactobacillus*) and inhibiting harmful microbes (e.g., *norank_f_Muribaculaceae*) (16). However, to the best of our knowledge, little is known regarding the action of DMY on growth performance and intestinal epithelial functions in weaned pigs infected with ETEC. Hence, this study aimed to assess the effectiveness of dietary DMY supplementation on intestinal inflammation and epithelium injury in weaned pigs induced by ETEC, and the potential mechanisms of action were also investigated.

## 2 Materials and methods

### 2.1 Animal experimental design

All animal procedures were approved by the Committee on Animal Care Advisory of Sichuan Agricultural University (authorization number SICAU-2022-014). All experiment procedures were carried out in accordance with the guidelines for the Care and Use of Laboratory Animals. In total, 24 healthy barrows weaned on their 21st day and with an average body weight of 7.84 ± 0.08 kg were individually housed in a metabolism cage (0.7 m × 1.5 m × 1.5 m) and allowed to acclimatize to the study condition for 3 days. All pigs were randomly allocated to three groups (with eight replicates in each group and one pig in each replicate). Pigs in the CON and ECON groups were fed with a basal diet, and those in the EDMY group were fed the basal diet added with 300 mg kg^−1^ DMY (Guangzhou Nuacid Co., Ltd., Guangzhou, China) for 21 days. On day 19 (i.e., after the adaptation period), pigs in the ECON and EDMY groups were orally administered 100 mL of LB culture containing approximately 2.5 × 10^9^ cfu mL^−1^ *E. coli* K88 (hereafter referred to as ETEC; serotype O149: K91: K88ac), while pigs in the CON group were administered equivalent amount of sterile LB medium. The basal diet (Table 1) was formulated to meet the swine nutrient requirements recommended by the National Research Council (NRC, 2012). Fresh feed and water were provided *ad libitum* to pigs throughout the experimental period. The ambient temperature of 26°C ± 1.5°C and relative humidities of 65% ± 5% were maintained. The ETEC F4+ (serotype O149:K91, K88ac) used in the challenge study was purchased from China Veterinary Culture Collection Center.

**Table 1:** Composition and nutrient levels of the experimental diets (air-dry basis, %).

Ingredient	Content (%)
Corn, 7.8% CP	26.73
Extruded corn, 8.2% CP	25.45
Soybean meal, 44.2% CP	9.50
Extruded soybean, 51.1% CP	10.50
Soybean oil	2.00
Fishmeal, 62.5%	4.00
Whey powder	7.00
Soybean protein concentrate	8.00
Sucrose	4.00
Stone powder	0.90
L-Lys HCl, 78%	0.47
DL-Methionine, 99%	0.15
L-Threonine, 98.5%	0.13
L-Tryptophan, 98%	0.03
Choline chloride, 50%	0.10
Calcium phosphate	0.50
NaCl	0.30
Mineral premix^a^	0.20
Vitamin premix^b^	0.04
Total	100
**Nutrient levels**^c^	
Digestible energy, Mcal/kg	3.55
Crude protein, %	19.80
Calcium, %	0.92
Available phosphorus, %	0.37
Lysine, %	1.41
Methionine, %	0.47
Methionine + cysteine, %	0.75
Threonine, %	0.79
Tryptophan, %	0.22

a The mineral premix provided the following per kg of diet: Fe (FeSO_4_·H_2_O), 120 mg; Cu (CuSO_4_·5H_2_O), 6 mg; Zn (ZnSO_4_·H_2_O), 100 mg; Mn (MnSO_4_·H_2_O), 40 mg; I (KI), 0.3 mg; Se (Na_2_SeO_3_), 0.3 mg.

b The vitamin premix provided the following per kg of diet: vitamin A, 6000 IU; vitamin D_3_, 400 IU; vitamin E, 10.0 IU; vitamin K_3_, 2.0 mg; vitamin B_1_, 0.8 mg; vitamin B_2_, 6.4 mg; vitamin B_6_, 2.4 mg; vitamin B_12_, 0.012 mg; niacin, 30 mg; pantothenic acid, 15 mg; folic acid, 0.75 mg; biotin, 0.1 mg.

c The diet was formulated based on the recommendation of the NRC 2012.

### 2.2 Sample collection

From days 19 to 21 of the experiment, the fresh fecal samples were collected immediately after excretion from pigs in each group. After collection, the daily excreta of each pig were weighed, and 10 mL of a 10% H_2_SO_4_ solution was added to each 100 g of wet fecal sample, and subsequently stored in a sealed plastic bag at −20°C. At the end of the experiment, all fecal samples of each pig were thawed at room temperature, mixed thoroughly, and then dried at 70°C for 48 h. After being crushed on a 1-mm screen, these samples were stored at −20°C for chemical analyses including dry matter (DM), crude protein (CP), ether extract (EE), ash, and gross energy (GE).

At 8:00 a.m. on day 21, blood samples were collected from the jugular vein of overnight-fasted piglets into non-heparinized vacuum tubes. The serum samples were prepared by centrifuging blood samples at 3,500 × g at 4°C for 20 min and then stored in a −20°C refrigerator until analysis. Subsequently, the pigs were euthanized with an intravenous injection of sodium pentobarbital (200 mg/kg BW) and followed by an exsanguination protocol. After euthanization, the duodenum, jejunum, and ileum samples of each pig were separated from the exposed abdomen and washed with ice-cold PBS. The mid segments of the duodenum, jejunum, and ileum, approximately 3 cm in length, were harvested and fixed in 4% paraformaldehyde solution for intestinal morphology. Other duodenum, jejunum, and ileum (approximately 4 cm in length) tissue samples were collected, snap-frozen, and stored at −80°C for real-time polymerase chain reaction (RT-PCR) analyses. The remaining segments of the duodenum, jejunum, and ileum were opened longitudinally, washed with ice-cold PBS, and gently scraped with a sterile glass microscope slide at 4°C to obtain mucosa samples. The mucosa samples were immediately snap-frozen in liquid nitrogen and stored at −80°C until further analysis of related enzyme activities. In addition, digesta of the colon and cecum was immediately placed in liquid nitrogen and stored at −80°C for analysis of microbial DNA and short-chain fatty acid (SCFA) concentration analyses.

### 2.3 Measurement of growth performance

Each pig’s body weight (BW) was monitored on days 1, 19, and 21 after 12-h fasting in the morning at 8:00, and feed intake and waste feed were collected and weighed daily. The average daily gain (ADG), average daily feed intake (ADFI), and the feed: gain ratio (F/G) were then calculated.

### 2.4 Determination of diarrhea rate

During the challenge period, the feces of the pigs were observed daily, and the diarrhea rate was calculated according to the formula, diarrhea rate (%) = (number of pigs with diarrhea within a treatment × total observational days) / (number of pigs × total observational days) × 100%. The incidence of diarrhea was defined in accordance with the following standards: fecal score of 0 (normal); fecal score of 1 (normal feces); fecal score of 2 (moist feces), fecal score of 3 (mild diarrhea), fecal score of 4 (severe diarrhea), and fecal score of 5 (watery diarrhea) in all the experiments. The occurrence of diarrhea was defined as maintaining fecal scores of 2 or 3 for two consecutive days.

### 2.5 Determination of the organ index

Organ index (%) was calculated as the organ fresh weight (kg)/piglet weight (kg) × 100% before slaughter.

### 2.6 Apparent total tract digestibility

The apparent total tract digestibility (ATTD) was determined according to the endogenous indicator acid-insoluble ash (AIA) standard method (GB/T 23742–2009). The procedures utilized for the determination of nutrient composition were conducted with the international standard methods described by the AOAC International (17), including dry matter (930.15; AOAC), crude protein (930.15; AOAC), crude fat (920.39; AOAC), and crude ash (942.05; AOAC). For calculating the ATTD of the nutrients, we used the following formula: ((100-A1 × F2/A2 × F1) × 100). A1: digesta nutrient; A2: digesta AIA; F1: diet AIA; F2: digesta AIA.

### 2.7 Serum parameter measurements

The concentrations of serum total protein (TP), albumin (ALB), alkaline phosphatase (AKP), glutamic oxaloacetic transaminase (GOT), glutamic pyruvic transaminase (GPT), total cholesterol (TC), glucose (GLU), triglyceride (TG), and urea (UREA) were gaged using an automatic biochemical analyzer (Olympus, Shanghai, China). Immunoglobulin A (IgA), including IgA, immunoglobulin G (IgG), and immunoglobulin M (IgM), were determined using a multi-mode microplate reader (SpectraMax M2, Molecular Devices, Sunnyvale, CA, United States) following the procedures outlined by the corresponding commercially available swine Enzyme-Linked Immunosorbent Assay (ELISA) kits (Jiangsu Meimian Industrial Co., Ltd., Yancheng, China). The minimum detection limits were 1 μg/mL, 12 μg/mL, and 1.2 μg/mL, respectively, and the intra-assay coefficients of variation (CV) were 6.3, 7.3, and 6.7%, and inter-assay CV were 10.2, 9.7, and 12.3%, respectively. The standards provided in the kits were used to generate standard curves for quantification and each test was run in duplicate.

### 2.8 Intestinal morphology measurement

For the intestinal morphology study, approximately 3 cm segments isolated from mid-duodenum, mid-jejunum, and mid-ileum were immobilized with 4% paraformaldehyde solution for 24 h and then embedded in paraffin. After cutting the paraffin-coated intestinal samples into sections of approximately 3 μm, they were transferred to a 70% ethanol solution for dehydration. Afterward, the samples were wax-embedded on slides and stained with hematoxylin and eosin. Each section was put on a slide for intestinal morphology observation by using an Olympus CK40 inverted phase-contrast microscope equipped with the AxioVision software. The villus height (VH) was measured from the tip to the villi–crypt junction, and the crypt depth (CD) was measured from the villi base to the lowest point of the CD; 20 well-orientated and intact villi and crypts from each segment were counted to evaluate intestinal morphology.

### 2.9 Intestinal enzyme activities

The concentrations of sucrase, lactase, maltase, and alkaline phosphatase (AKP) in intestinal mucosa were evaluated in accordance with the instructions of the respective kits (Nanjing Jiancheng Bioengineering Institute). Briefly, approximately 100 mg of each thawed intestinal mucosa sample extracted from the duodenum, jejunum, and ileum was homogenized with a precooled 0.9% saline and then centrifuged at 3,000 × g, 4°C for 15 min. The supernatant was assayed for protein content in accordance with the Bradford method (18), following the sucrase, lactase, maltase, and AKP activities were measured in triplicate on a spectrophotometer. The results were normalized to protein concentration and expressed as U/mg protein.

### 2.10 Gene expression analysis

For RNA extraction, approximately 0.1 g of each frozen sample isolated from duodenum, jejunum, and ileum was homogenized with 1 mL of RNAiso Plus reagent (TaKaRa, Dalian, China) to extract the total RNA according to the manufacturer’s instructions. After analyzing integrity using 1.0% agarose gel, RNA purity and concentration were quickly determined on a spectrophotometer (NanoDrop-ND2000, Thermo Fisher Scientific, Inc., Waltham), of which OD260/OD280 ratio ranged from 1.8 to 2.0 were deemed appropriate. Subsequently, 1.0 μg of total RNA was reverse-transcribed into complementary DNA (cDNA) for RT-PCR using the PrimeScript™ RT reagent kit with gDNA Eraser (Takara Biotechnology Co., Ltd., Dalian, China).

As shown in Table 2, the primers were designed with Primer 5.0 software to amplify target gene fragments by performing q-PCR. The q-PCR was performed with the SYBR^®^ Green PCR I PCR reagents (Takara Bio Inc., Dalian, China) on a CFX96 Real-Time PCR Detection System (Bio-Rad Laboratories, Hercules, CA, United States). All cDNA samples were detected in triplicate. Each q-PCR consisted of 1 μL of cDNA, 5 μL of SYBR Green JumpStart Taq ReadyMix (1×), 0.2 μL of ROX Reference Dye II (50×), 0.4 μL of each primer, and 3 μL of diethyl pyrocarbonate-treated water in a total volume of 10 μL. The protocol used in q-PCR was as follows: 95°C for 30 s, followed by 40 cycles: at 95°C for 5 s and 60°C for 34 s. The generated gene-specific amplification products were confirmed by melting curve analysis after each real-time quantitative PCR assay. The housekeeping gene glyceraldehyde-3-phosphate dehydrogenase (GAPDH) was used to standardize the mRNA expression level of target genes, which was calculated based on the 2^–ΔΔCt^ method (19).

**Table 2:** Primers used for real-time PCR analysis.

Gene^a^	GenBank no.	Primer sequence (5′ to 3′)^b^	AT, °C^c^	Product size, bp
*β-actin*	XM_003124280.5	F: TGGAACGGTGAAGGTGACAGC	62	177
		R: GCTTTTGGGAAGGCAGGGACT		
*GLUT-2*	NM_001097417.1	F: TGGAATCAGCCAACCTGTTT	58	165
		R: ACAAGTCCCACCGACATGA		
*CAT-1*	XM_021065165.1	F: TGCCCATACTTCCCGTCC	60	192
		R: GGTCCAGGTTACCGTCAG		
*FATP-1*	XM_021076151.1	F: GGAGTAGAGGGCAAAGCAGG	61	208
		R: AGGTCTGGCGTGGGTCAAAG		
*ZO-1*	XM_021098896.1	F: CAGCCCCCGTACATGGAGA	61	114
		R: GCGCAGACGGTGTTCATAGTT		
*Occludin-1*	XM_005672525.3	F: CTACTCGTCCAACGGGAAAG	59	158
		R: ACGCCTCCAAGTTACCACTG		
*Claudin-1*	NM_001244539.1	F: TCTTAGTTGCCACAGCATGG	59	106
		R: CCAGTGAAGAGAGCCTGACC		

a GLUT-2, glucose transporter 2; CAT-1, cationic amino acid transporter 1; FATP-1, fatty acid transport protein 1; ZO-1, zonula occludens-1.

b F, forward primer; R, reverse primer.

c AT, annealing temperature.

### 2.11 Intestinal microbiological analysis

The digesta samples (approximately 200 mg) isolated from the caecum and colon in individual pigs were used to extract total microbial DNA using the Stool DNA Kit (Omega Bio-Tek, Doraville, CA, United States) according to the manufacturer’s instructions. Based on the sequences downloaded from the National Center for Biotechnology Information (GenBank), the primers and probes listed in Table 3 were designed to amplify the reactions of total bacteria, *E. coli*, *Lactobacillus*, *Bifidobacterium,* and *Bacillus* on a CFX96 Real-Time PCR system (Bio-Rad Laboratories, Inc., Hercules, CA).

**Table 3:** Sequence of primers and probes for selected bacteria.

Gene	Primer sequence (5′ to 3′)^a^	AT, °C^b^	Product size, bp
Total bacteria	F: ACTCCTACGGGAGGCAGCAG	60	200
	R: ATTACCGCGGCTGCTGG		
*Lactobacillus*	F: GAGGCAGCAGTAGGGAATCTTC	60	126
	R: CAACAGTTACTCTGACACCCGTTCTTC		
	P: AAGAAGGGTTTCGGCTCGTAAAACTCTGTT		
*Escherichia coli*	F: CATGCCGCGTGTATGAAGAA	60	96
	R: CGGGTAACGTCAATGAGCAAA		
	P: AGGTATTAACTTTACTCCCTTCCTC		
*Bifidobacterium*	F: CGCGTCCGGTGTGAAAG	60	121
	R: CTTCCCGATATCTACACATTCCA		
	P: ATTCCACCGTTACACCGGGAA		
*Bacillus*	F: GCAACGAGCGCAACCCTTGA	60	92
	R: TCATCCCCACCTTCCTCCGGT		
	P: CGGTTTGTCACCGGCAGTCACCT		

a F, forward primer; R, reverse primer.

b AT, annealing temperature.

More precisely, total bacteria were detected by the reaction, which runs in a volume of 25 μL with 12.5 μL SYBR Premix Ex Taq (2×), 1 μL of each primer (100 nmol/L), 1 μL 50 × ROX Reference Dye*3, 7.5 μL of RNase-Free ddH_2_O, and 2 μL template DNA. The SuperReal PreMix (Probe) kit (Tiangen Biotech Co., Ltd., Beijing, China) was used for *Lactobacillus*, *E. coli*, *Bacillus,* and *Bifidobacterium* detection. Each reaction was run in a volume of 25 μL with 12.5 μL Super Real PreMix (2×), 1 μL of each primer (100 nmol/L), 1 μL probe (100 nmol/L), 1 μL 50 × ROX Reference Dye*3, 6.5 μL of RNase-Free ddH_2_O, and 2 μL DNA. All reaction cycling protocols consisted of enzyme activation and denaturation at 95°C for 15 min; 40 cycles of 95°C for 3 s and 60°C for 30 s, followed by dissociation at 60–95°C with 0.5°C increments every 1 s. The cycle threshold (Ct) values and baseline settings were determined by automatic analysis settings, and the copy numbers of the target group for each reaction were calculated from the standard curves, which were generated by constructing standard plasmids by a 10-fold serial dilution of plasmid DNA (1 × 10^1^ to 1 × 10^9^ copies/μL).

### 2.12 Analysis of SCFAs

The SCFAs in digesta were determined using gas–liquid chromatography according to the previously method described (20). Briefly, approximately 0.5 g of thawed digesta was diluted with 2 mL of sterile Milli-Q water. After being vortexed, the entire sample was centrifuged at 12,000 × g for 10 min to obtain the suspension liquid. Then, a sample of 25% metaphosphoric acid solution was combined in a 9:1 ratio and centrifuged at 12,000 × g again for 10 min after being placed at 4°C for 30 min. Finally, a 0.45-mm filter membrane was used to filter the supernatant after it was aspirated with a syringe. The gas chromatographic system (VARIAN CP-3800, America) equipped with a polyethylene glycol-packed column with an inner diameter of 0.32 mm and 30 m length, and 0.25 μm film thickness, was used to separate and quantify the SCFAs (e.g., acetate, propionate, and butyrate). After each injection sample (1 μL) entered the column, the column temperature rose from the initial temperature of 70°C to 150°C within 3 min, while the temperature of the injector and detector reached 250°C.

### 2.13 Statistical analysis

All data were subjected to a one-way analysis of variance for a completely randomized design using the general linear model procedure of SPSS 24.0 (SPSS, Inc.), with each pig representing one experimental unit. Statistical differences among treatments were separated using Tukey’s multiple-range test. The results are expressed as with their standard errors. Statistical significance was set at *p* < 0.05 and 0.05 < *p* < 0.10, indicating a trend.

## 3 Results

### 3.1 Effect of DMY on growth performance, organ index, and nutrient digestibility in weaned pigs upon ETEC challenge

Before the ETEC challenge, DMY treatment had no effect (*p* > 0.05) on initial weight, final weight, and F/G (Table 4). During days 19–21, ETEC significantly reduced (*p* < 0.05) the final weight and ADG in the weaned pigs of the ECON group compared to the pigs of the CON group. In particular, DMY supplementation significantly increased (*p* < 0.05) the final weight and ADG but decreased (*p* < 0.05) the diarrhea incidence and kidney index in the weaned pigs of the EDMY group compared to the pigs of the ECON group (Table 5). Compared to the CON and ECON groups, the EDMY group showed a significant increase in the apparent digestibility of DM, EE, GE, and ash (Table 6).

**Table 4:** Effect of dietary DMY supplementation on the growth performance of weaned pigs upon ETEC challenge.^1^

Items	Treatments^2^			SEM	*p*-value
	CON	ECON	EDMY		
**1–18 d**					
Initial weight, kg	7.88	7.72	7.89	0.08	0.75
Final weight, kg	12.01	11.60	12.95	0.30	0.67
ADFI, g/d	374.14	363.00	444.20	21.43	0.09
ADG, g/d	222.04	219.44	281.39	15.80	0.07
F/G	1.69	1.69	1.61	0.05	0.32
Diarrhea rate, %	29.61	28.73	28.33	2.31	0.71
**19–21 d**					
Initial weight, kg	11.86	11.76	12.95	0.30	0.54
Final weight, kg	13.03^a^	12.04^b^	13.78^a^	0.34	<0.01
ADFI, g/d	353.09	315.71	385.3	17.21	0.28
ADG, g/d	209.72^ab^	95.24^b^	274.1^a^	27.19	0.02
F/G	1.72	–	1.48	0.57	0.10
Diarrhea rate, %	12.50^c^	45.83^a^	27.5^b^	1.70	0.03

ADG, average daily gain; ADFI, average daily feed intake; FC, feed conversion ratio.

1 Data are means of eight replicates per treatment.

2 CON, pigs were fed with a basal diet; ECON, pigs were fed with a basal diet and challenged by ETEC; EDMY, pigs were fed with a DMY-containing diet and challenged by ETEC.

^a,b^Within a row, values with different superscript letters differ (*p* < 0.05).

**Table 5:** Effects of dietary DMY supplementation on organ index of weaned piglets upon ETEC challenge.^1^

Items	Treatments^2^			SEM	*p*-value
	CON	ECON	EDMY		
Heart index, %	0.46	0.48	0.46	0.01	0.79
Liver index, %	2.58	2.60	2.71	0.07	0.71
Spleen index, %	0.16	0.17	0.19	0.01	0.24
Lung index, %	1.43	1.64	1.57	0.06	0.41
Kidney index, %	0.53^ab^	0.59^a^	0.51^b^	0.02	0.05

1 Data are means of eight replicates per treatment.

2 CON, pigs were fed with a basal diet; ECON, pigs were fed with a basal diet and challenged by ETEC; EDMY, pigs were fed with a DMY-containing diet and challenged by ETEC.

^a,b^Within a row, values with different superscript letters differ (*p* < 0.05).

**Table 6:** Effect of dietary DMY supplementation on nutrient digestibility of weaned pigs upon ETEC challenge^1^.

Items	Treatments^2^			SEM	*p*-value
	CON	ECON	EDMY		
DM, %	87.29^b^	89.07^b^	92.20^a^	0.59	<0.01
EE, %	81.46^b^	84.60^ab^	88.28^a^	1.04	0.02
GE, %	87.29^b^	89.50^b^	92.15^a^	0.63	<0.01
Ash, %	62.93^b^	66.99^b^	77.62^a^	1.71	<0.01
CP, %	82.91	85.28	87.59	1.09	0.18

DM, dry matter; CP, crude protein; EE, ether extract.

1 Data are means of 8 replicates per treatment.

2 CON, pigs were fed with a basal diet; ECON, pigs were fed with a basal diet and challenged by ETEC; EDMY, pigs were fed with a DMY-containing diet and challenged by ETEC.

^a,b^Within a row, values with different superscript letters differ (*p* < 0.05).

### 3.2 Effect of DMY on serum biochemical indices and immunoglobulins in weaned pigs upon ETEC challenge

**Figure 1:**
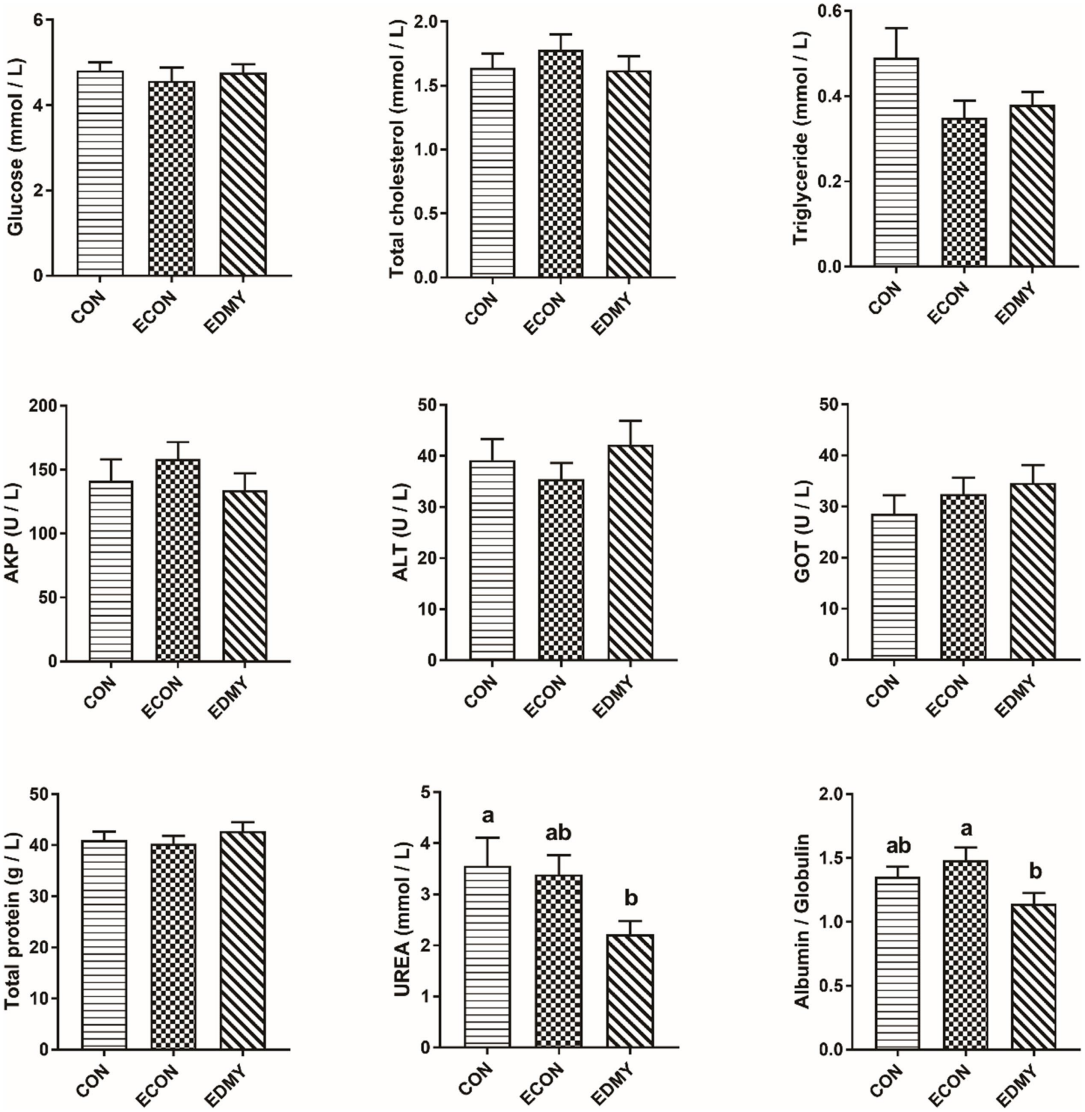
Effect of dietary DMY supplementation on plasma biochemical indices concentrations of weaned pigs upon ETEC challenge. Values are means ± SEM, (*n* = 8). CON, pigs were fed with a basal diet; ECON, pigs were fed with a basal diet and challenged by ETEC; EDMY, pigs were fed with a DMY containing diet and challenged by ETEC. AKP, alkaline phosphatase; ALT, alanine aminotransferase; GOT, glutamic oxalacetic transaminase.

**Figure 2:**
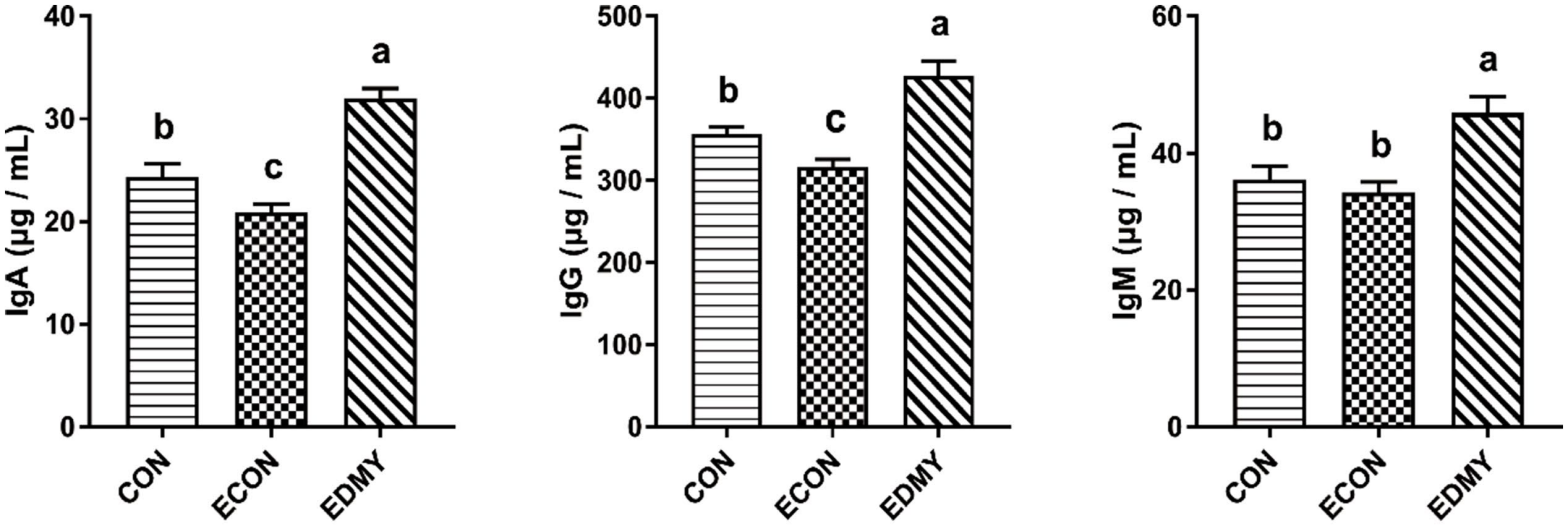
Effect of dietary DMY supplementation on plasma Immunoglobulin concentrations of weaned pigs upon ETEC challenge. Values are means ± SEM, (*n* = 8). CON, pigs were fed with a basal diet; ECON, pigs were fed with a basal diet and challenged by ETEC; EDMY, pigs were fed with a DMY containing diet and challenged by ETEC. IgA, immunoglobulin A; IgG, immunoglobulin G; IgM, immunoglobulin M.

As shown in Figure 1, the ETEC challenge significantly decreased (*p* < 0.05) concentrations of immunoglobulins such as IgA and IgG in the weaned pigs of the ECON group compared to the pigs of the CON group. However, DMY supplementation not only decreased (*p* < 0.05) albumin/globulin ratio but also elevated levels of serum levels of IgA, IgG, and IgM in the weaned pigs of the EDMY group compared to the pigs of the ECON group (Figure 2).

### 3.3 Effect of DMY supplementation on intestinal morphology and mucosal enzyme activity in weaned pigs upon ETEC challenge

**Figure 3:**
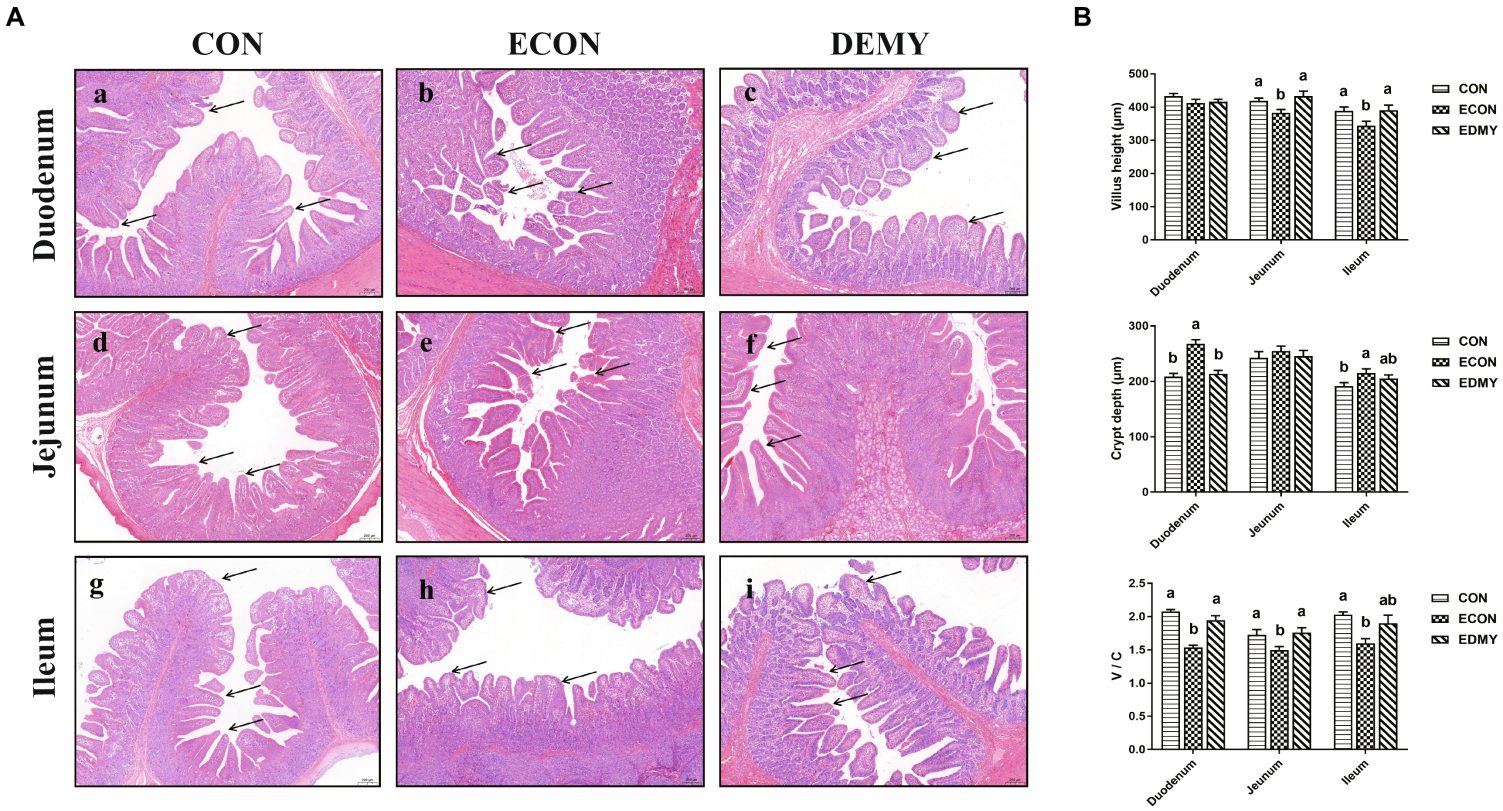
Effect of dietary DMY supplementation on small intestinal morphology of weaned pigs upon ETEC challenge (H&E; × 40). **(A)** Representative photomicrographs of villi in the duodenum (a−c), jejunum (d−f) and ileum (g−i) from pigs in the CON, ECON, and EDMY groups, respectively. **(B)** Villus height, crypt depth, and villus height to crypt depth ratio of the duodenum, jejunum, and ileum of piglets in the CON, ECON, and EDMY groups, respectively. CON, pigs were fed with a basal diet; ECON, pigs were fed with a basal diet and challenged by ETEC; EDMY, pigs were fed with a DMY containing diet and challenged by ETEC.

The ETEC challenge significantly decreased (*p* < 0.05) the villus height of the jejunum and ileum of the weaned pigs of the ECON group compared to those in pigs of the CON group. However, DMY treatment significantly increased (*p* < 0.05) the villus height of the jejunum and ileum of the weaned pigs of the EDMY group compared to those in pigs of the ECON group. For crypt depth, DMY treatment significantly decreased (*p* < 0.05) the crypt depth in the duodenum of the weaned pigs of the EDMY group compared to that in pigs of the ECON group. Additionally, although the ETEC challenge significantly decreased (*p* < 0.05) ratio of V: C in the duodenum and jejunum of the weaned pigs of the ECON group compared to those in pigs of the CON group, DMY treatment eliminated this effect (Figure 3).

In Table 7, DMY treatment increased the duodenal mucosa AKP, sucrase, and maltase activities, jejunal mucosa lactase and sucrase activities, and ileal mucosa sucrase activity in the weaned pigs of the EDMY group compared to the pigs of the ECON group (*p* < 0.05). Moreover, the mucosa activities of sucrase and maltase in the duodenum were higher (*p* < 0.05) in the EDMY group than those in the ECON group.

**Table 7:** Effect of dietary DMY supplementation on mucosal enzyme activity of small intestine in weaned piglets upon ETEC challenge.^1^

Items	Treatments^2^			SEM	*p-*value
	CON	ETEC	ETECPE		
**Duodenum**					
AKP, U/g protein	8.12^a^	6.17^b^	7.82^a^	0.40	0.04
Sucrase, U/mg protein	162.35^b^	160.14^b^	202.50^a^	15.54	0.02
Lactase, U/mg protein	180.26	166.82	177.66	16.18	0.68
Maltase, U/mg protein	216.71^b^	221.71^b^	305.47^a^	27.13	<0.01
**Jejunum**					
AKP, U/g protein	11.25^a^	8.52^b^	9.51^ab^	0.43	0.05
Sucrase, U/mg protein	379.77^a^	303.82^b^	370.20^a^	23.22	<0.01
Lactase, U/mg protein	307.45^a^	227.72^b^	320.11^a^	24.38	<0.01
Maltase, U/mg protein	774.16	661.24	624.68	80.24	0.17
**Ileum**					
AKP, U/g protein	4.12	3.59	3.73	0.31	0.32
Sucrase, U/mg protein	51.79^ab^	38.33^b^	54.62^a^	5.74	0.02
Lactase, U/mg protein	74.80	62.10	50.27	12.36	0.16
Maltase, U/mg protein	122.01	102.69	102.40	10.21	0.11

AKP, alkaline phosphatase.

1 Data are means of eight replicates per treatment.

2 CON, pigs were fed with a basal diet; ECON, pigs were fed with a basal diet and challenged by ETEC; EDMY, pigs were fed with a DMY-containing diet and challenged by ETEC.

^a,b^Within a row, values with different superscript letters differ (*p* < 0.05).

### 3.4 Effect of DMY supplementation on expressions of critical genes involved in intestinal epithelium functions

**Figure 4:**
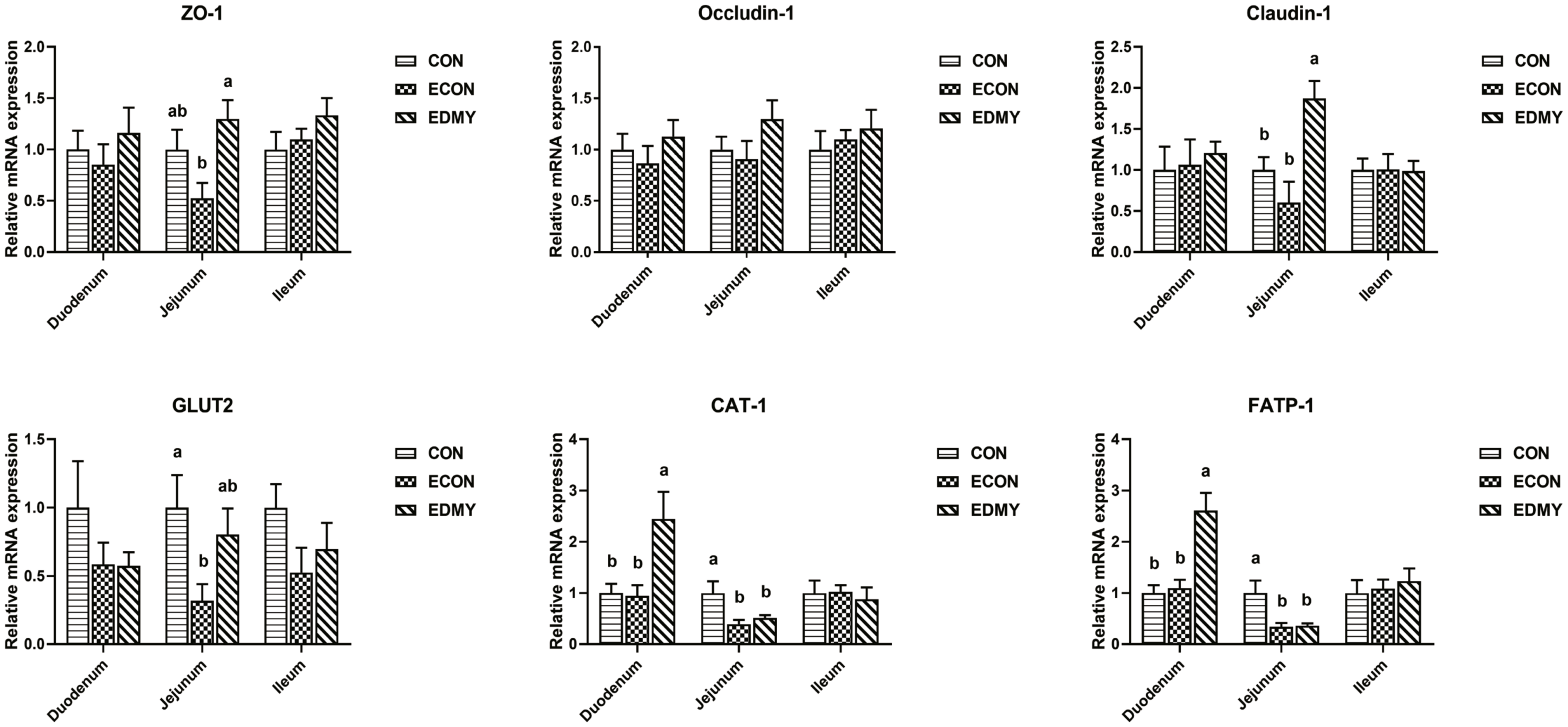
Relative expression levels of critical genes involved in the intestinal barrier functions. Values are means ± SEM, (*n* = 8). Within a panel, bars labeled with different superscript letters significantly different at *P* < 0.05. CON, pigs were fed with a basal diet; ECON, pigs were fed with a basal diet and challenged by ETEC; EDMY, pigs were fed with a DMY containing diet and challenged by ETEC. ZO-1, zonula occludens-1; GLUT2, glucose transporter-2; CAT1, cationic amino acid transporter-1; FATP1, Fatty acid transport protein-1.

As shown in Figure 4, the ETEC challenge significantly decreased (*p* < 0.05) the expression levels of GLUT2, CAT-1, and FATP-1 in the jejunum of the weaned pigs of the ECON group compared to those in pigs of the CON group. However, DMY supplementation not only elevated the mRNA expression levels of ZO-1 and Claudin-1 in the jejunum but also elevated the mRNA expression levels of CAT-1 and FATP-1 in the duodenum of the EDMY group compared to those of the ECON group (*p* < 0.05).

### 3.5 Effect of DMY supplementation on intestinal microbial populations and metabolites in weaned pigs upon ETEC challenge

**Figure 5:**
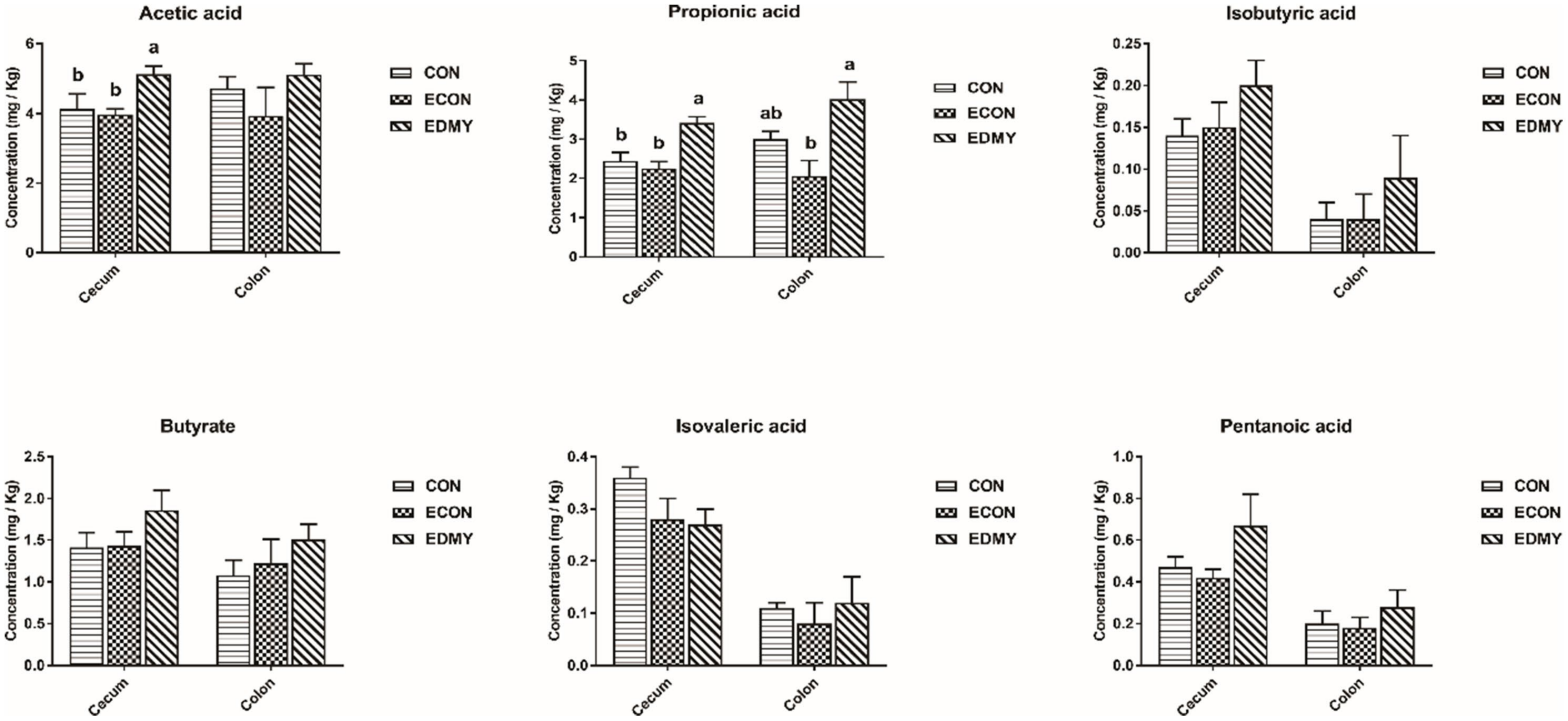
Effect of dietary DMY supplementation on concentrations of VFAs in digesta of cecum and colon in weaned pigs upon ETEC challenge. Values are means ± SEM, (*n* = 8). CON, pigs were fed with a basal diet; ECON, pigs were fed with a basal diet and challenged by ETEC; EDMY, pigs were fed with a DMY containing diet and challenged by ETEC.

In Table 8, the ETEC challenge significantly increased (*p* < 0.05) the abundance of *Escherichia coli* in the cecum and colon as well as total bacteria in the colon of weaned piglets in the ECON group compared to those in the CON group. DMY supplementation significantly decreased (*p* < 0.05) the abundance of *Escherichia coli* in the colon of weaned piglets in the EDMY group compared to that in the ECON group. As shown in Figure 5, DMY supplementation significantly elevated (*p* < 0.05) the cecal and colonic concentrations of acetic acid and propanoic acid in weaned piglets of the EDMY group compared to those of the ECON group.

**Table 8:** Effect of dietary DMY supplementation on intestinal bacteria in digesta of cecum and colon in weaned piglets upon ETEC challenge.^1^

Items	Treatments^2^			SEM	*p*-value
	CON	ECON	EDMY		
**Cecum**					
Total bacteria, copies/g	11.09	10.98	10.94	0.03	0.10
*Escherichia coli*, copies/g	8.21^a^	10.11^a^	8.53^b^	0.26	0.03
*Lactobacillus*, copies/g	8.59	8.77	8.36	0.11	0.33
*Bifidobacterium*, copies/g	7.03	6.83	7.37	0.24	0.68
*Bacillus*, copies/g	9.24	9.44	9.64	0.08	0.15
**Colon**					
Total bacteria, copies/g	11.18^b^	11.45^a^	11.31^ab^	0.05	0.03
*Escherichia coli*, copies/g	8.84^b^	10.52^a^	9.34^ab^	0.27	<0.01
*Lactobacillus*, copies/g	8.70	8.49	8.94	0.15	0.46
*Bifidobacterium*, copies/g	7.48	7.25	7.77	0.26	0.73
*Bacillus*, copies/g	9.59	9.41	9.71	0.07	0.18

1 Data are means of eight replicates per treatment.

2 CON, pigs were fed with a basal diet; ECON, pigs were fed with a basal diet and challenged by ETEC; EDMY, pigs were fed with a DMY-containing diet and challenged by ETEC.

^a,b^Within a row, values with different superscript letters differ (*p* < 0.05).

## 4 Discussion

It has been shown that ETEC can cause intestine disturbance and an increase in diarrhea rate and F/G of piglets, thereby establishing a feasible model of intestinal infection (5, 6, 21). DMY has been extensively studied as it possesses multiple health-promoting biological and pharmacological properties, of which antioxidative ability (12), anti-inflammatory (11), immune-enhancing (12), and intestinal microbiota regulating biological activities (13) may be pertinent to improving growth performance of animals, especially during the weaning period. A previous study indicated that dietary supplementation of DMY at 500 mg/kg decreased F/G in growing-finishing pigs (12). In contrast, the current results suggested that oral administration of DMY alone did not show beneficial effects on F/G and diarrhea rate of piglets during days 1–18 (pre-challenge). Given that numerous plant-derived compounds are more effective when livestock animals suffer from pathogenic pressure (22–24). Accordingly, the positive effect of DMY was obvious under the condition of the ETEC challenge. In this study, we found that dietary DMY not only reversed the negative alternations in diarrhea rate caused by ETEC and therefore improved the ADG and F/G, but also increased the digestibility of DM, Ash, EE, and GE in the ETEC-challenged pigs, suggesting a beneficial effect of DMY supplementation on the intestinal health of weaned pigs.

The metabolic and health status of animal bodies or organs could be reflected by the blood biochemical parameters. In the male Sprague–Dawley rat study, the result showed that feeding 5 μg/kg body weight DMY decreased the level of blood urea nitrogen, which is a marker of kidney injury (25). Consistent with the previous report, this study found that dietary DMY had decreased serum UREA level and kidney index in the ETEC-challenged pigs, suggesting DMY alleviated the symptoms associated with the infection of weaned pigs challenged with ETEC. The albumin, globulin, and A/G ratio can be used as an indicator to indicate the situation of protein synthesis and nutritional status *in vivo*, and it is worth noting that a large number of immunoglobulins constitute an important source of globulin (26, 27). In this study, dietary DMY downregulated the plasma A/G ratio and globulin, indicating that DMY may influence the immune status of piglets. Generally, the levels of Immunoglobulins such as IgG, IgM, and IgA are used as indicators of immunological status, which play a critical role in clearing particular bacteria or viruses (28). In the present study, DMY significantly elevated the serum IgA, IgG, and IgM concentrations in the ETEC-challenged pigs, which is consistent with a previous study (12), indicating an enhanced immunity upon DMY supplementation.

The small intestine is of critical importance for nutrient digestion and absorption, and its villous height and V/C ratio have been considered as integrated morphological structure indicators to evaluate intestinal health, such as intestinal barrier integrity, nutrient digestion, and absorption capacity (29). Recently, several studies have confirmed that ETEC strains produce enterotoxins that act on the enterocytes, leading to the secretion of fluids and electrolytes, which ultimately results in the impaired intestinal barrier and function (4–6). Along the same lines, we found that the ETEC challenge decreased the duodenal and ileal villus height, and significantly reduced the ratio of V/C in the duodenum and jejunum, indicating injury of the intestinal epithelium. However, dietary DMY supplementation attenuated the intestinal injury by increasing the villus height and the ratio of V/C. Changes in the intestinal morphology are usually accompanied by the mucosal enzyme activities expressed in the brush border, which can reflect the function of intestinal epithelium (30). In the present study, dietary DMY supplementation not only elevated duodenal the activities of sucrase, maltase, and AKP but also elevated the jejunal activity of AKP, sucrase, and lactase in the ETEC-challenged pigs. As important endogenous enzymes, sucrase, maltase, and lactase are considered an excellent marker enzyme involved in carbohydrate digestion (31). However, alkaline phosphatase (AKP) has received wide attention due to its important protective properties in the gut, including absorption of lipids, detoxification of bacterial lipopolysaccharide, and possible modulation of the gut microbiota (32). All the evidence suggested that dietary DMY has a protective effect on intestinal health.

To gain insights into the mechanisms underlying the DMY-regulated intestinal health, we further investigated the expression levels of several critical genes involved in intestinal epithelium functions. The occludin, ZO-1, and Claudin-1 were members of transmembrane barrier proteins, cytoplasmic scaffold proteins, and adhesion molecules proteins of tight junctions (TJs), respectively, which play a key role in maintaining intestinal permeability (33). However, *Escherichia coli* lipopolysaccharide-induced injury in the intestinal barrier by reducing the expression of TJs (34). In the present study, dietary DMY supplementation not only significantly elevated the expression levels of ZO-1 and Claudin-1 but also elevated the expression levels of nutrient transporters (CAT-1, and FATP-1) in the intestinal epithelium of ETEC-challenged pigs. CAT-1 is a small molecule protein that is responsible for the transportation of cationic amino acids across cell membranes (35). However, the FATP-1 is closely associated with the transportation of fatty acids (36). The elevated expressions of these nutrient transporters may contribute to the improved barrier integrity and functions of intestinal epithelium in piglet exposure to the ETEC challenge.

It has been reported that DMY inhibits the growth of *Staphylococcus aureus* by disrupting membrane integrity and decreasing activities of a few energy metabolism enzymes, total ATPase (37). Similarly, we unexpectedly found that dietary DMY selectively inhibits the growth of potential pathogenic bacterial species (*Escherichia coli*) in this study. Recently, increasing evidence suggested that a dysregulated or perturbed state of the gut microbiota increases the susceptibility of piglets to enteric pathogens (38). Although we did not find that DMY affected the abundance of *Lactobacillus*, *Bifidobacterium*, and *Bacillus* in this study, it is necessary to conduct more extensive microbiome research in the future to explore whether DMY can improve ETEC-induced microbiota disturbance. Meanwhile, SCFAs (e.g., acetic acid, propionic acid, and butyric acid), as metabolites of the intestinal microbiota have been increasingly focused on due to their effective antimicrobial properties (39–41). Moreover, SCFAs not only act as a substrate for energy production but also regulate cell proliferation, apoptosis, and immunity, thereby promoting the functional maturation of intestinal epithelial cells (42). In the present study, dietary DMY was able to elevate SCFA levels in digesta of ETEC-challenged pigs. These results were consistent with the trend of intestinal barrier integrity and nutrient digestion and absorption capacity in the present study.

## 5 Conclusion

Hence, this study confirmed the protective effect of DMY as a dietary supplement in alleviating ETEC-induced intestinal injury in weaning pigs. As metabolites of intestinal microbiota regulated by DMY, SCFAs protect the intestinal barrier and nutrient digestion and absorption capacity, which might be the potential mechanism of dietary DMY supplements to protect intestinal injury induced by ETEC exposure. In addition, this study will also provide a new perspective on the application of dietary DMY in swine production.

## Data availability statement

The raw data supporting the conclusions of this article will be made available by the authors, without undue reservation.

## Ethics statement

The animal studies were approved by the Committee on Animal Care Advisory of Sichuan Agricultural University (authorization number SICAU-2022-014). All experiment procedures were carried out in accordance with the guidelines for the Care and Use of Laboratory Animals. The studies were conducted in accordance with the local legislation and institutional requirements. Written informed consent was obtained from the owners for the participation of their animals in this study.

## Author contributions

KX: Formal analysis, Project administration, Software, Writing – original draft. JQ: Formal analysis, Project administration, Software, Writing – original draft. LD: Formal analysis, Writing – original draft. BY: Investigation, Methodology, Writing – review & editing. YuL: Investigation, Methodology, Writing – review & editing. ZH: Methodology, Writing – review & editing. XM: Methodology, Writing – review & editing. JY: Methodology, Writing – review & editing. PZ: Methodology, Writing – review & editing. HY: Methodology, Writing – review & editing. YaL: Methodology, Writing – review & editing. HL: Methodology, Writing – review & editing. JH: Conceptualization, Funding acquisition, Investigation, Resources, Writing – review & editing.
