# Corrigendum: Dihydromyricetin improves growth performance, immunity, and intestinal functions in weaned pigs challenged by enterotoxigenic *Escherichia coli*

**DOI:** 10.3389/fvets.2024.1481509

**Published:** 2024-09-17

**Authors:** Kunhong Xie, Jiawen Qi, Lili Deng, Bing Yu, Yuheng Luo, Zhiqing Huang, Xiangbing Mao, Jie Yu, Ping Zheng, Hui Yan, Yan Li, Hua Li, Jun He

**Affiliations:** ^1^Institute of Animal Nutrition, Sichuan Agricultural University, Chengdu, China; ^2^Key Laboratory for Animal Disease-Resistance Nutrition of China Ministry of Education, Chengdu, China; ^3^College of Veterinary Medicine, Sichuan Agricultural University, Chengdu, China

**Keywords:** *Escherichia coli*, immunity, DMY, intestinal epithelium, microbiota, weaned pigs

In the published article, there was an error in the **Funding** statement. The project number for the first project was incomplete, with a final number “0” missing. The Funding originally read:

“This study was supported by the National Key R&D Program of China (2023YFD130120), and The Innovation Team of Sichuan Province (SCCXTD-2024-8).”

The correct **Funding** statement appears below.

“This study was supported by The National Key R&D Program of China (2023YFD1301200), and The Innovation Team of Sichuan Province (SCCXTD-2024-8).”

The authors apologize for this error and state that this does not change the scientific conclusions of the article in any way. The original article has been updated.

